# 52 Endoscopy-associated Salmonella Infections: Nearly Forgotten but Not Gone

**DOI:** 10.1017/ash.2026.10487

**Published:** 2026-06-23

**Authors:** Dominique Balan, Katelynn Devinney, Portier Thomas, Alison Levin-Rector, Isatou Jobe, Elise Mantell, William Greendyke, Molly Kratz, Tristan McPherson, Nicole Burton

**Affiliations:** 1 New York City Department of Health & Mental Hygiene; 2 NYC Department of Health and Mental Hygiene; 3 New York City Department of Health and Mental Hygiene; 4 NYC Dept of Health and Mental Hygiene; 5 NYC DOHMH; 6 New York City Health Dept

## Abstract

**Background:** Carbapenem-resistant organisms (CRO) and Candida auris are multidrug-resistant organisms (MDRO) that can spread between patients and environments, posing a threat to health care systems, including acute care hospitals and nursing homes. However, their impact on non-nursing home congregate residential facilities, such as homeless shelters and assisted living facilities, is poorly understood. These facilities traditionally lack infection prevention and control (IPC) capacity as compared with health care facilities, have multi-occupancy rooms, and may have high censuses, all of which facilitate disease spread. **Methods:** To inform opportunities for public health intervention, clinical CRO and C. auris cases were enumerated from results reported to the New York City Health Department during 2019–2024. Patients’ geocoded addresses were matched to a list of non-nursing home congregate residential facilities. Cases were counted once during the study period, in alignment with the 2023 Council of State and Territorial Epidemiologists Carbapenemase-Producing Organisms and C. auris case definitions. CRO cases were counted separately for each organism/carbapenemase combination among patients where multiple combinations were reported. Cases’ demographic, isolate, and residential facility characteristics were summarized using univariate statistics. **Results:** During 2019–2024, 97% (11507/11858) of CRO and 100% of 1848 C. auris cases were reported with complete addresses. Of those, 471 (4%) CRO and 89 (5%) C. auris cases’ addresses matched a non-nursing home congregate residential facility at the time of diagnosis. Most CRO (63%) and C. auris (70%) cases were male. The median age at diagnosis for CRO cases was 64 years (interquartile range: 52–100 years) and for C. auris cases was 60 years (interquartile range: 51–69 years). Most laboratory tests originated from inpatient facilities for both CRO (65%) and C. auris (71%) cases. Among CRO and C. auris cases whose addresses matched to a congregate setting, the most common non-nursing home congregate facility types were group homes for people with intellectual and developmental disabilities (33% and 38%, respectively), shelters for people experiencing homelessness (25% and 26%), assisted living facilities (22% and 15%), and housing for people with mental health conditions (14% and 18%). **Conclusion:** More research is needed regarding CRO and C. auris in congregate settings. People residing in these environments may benefit from tailored approaches to MDRO case management. Enhanced surveillance by health departments in the form of routinized geocoding and address matching, which is low-cost and uses existing data sources, can identify facilities that could benefit from IPC support, education, and resource distribution.

